# The epidemiology of low back pain in primary care

**DOI:** 10.1186/1746-1340-13-13

**Published:** 2005-07-26

**Authors:** Peter M Kent, Jennifer L Keating

**Affiliations:** 1 School of Physiotherapy, La Trobe University, Melbourne, Victoria, Australia; 2 Physiotherapy, Monash University, Melbourne, Victoria, Australia

## Abstract

This descriptive review provides a summary of the prevalence, activity limitation (disability), care-seeking, natural history and clinical course, treatment outcome, and costs of low back pain (LBP) in primary care.

LBP is a common problem affecting both genders and most ages, for which about one in four adults seeks care in a six-month period. It results in considerable direct and indirect costs, and these costs are financial, workforce and social. Care-seeking behaviour varies depending on cultural factors, the intensity of the pain, the extent of activity limitation and the presence of co-morbidity. Care-seeking for LBP is a significant proportion of caseload for some primary-contact disciplines. Most recent-onset LBP episodes settle but only about one in three resolves completely over a 12-month period. About three in five will recur in an on-going relapsing pattern and about one in 10 do not resolve at all. The cases that do not resolve at all form a persistent LBP group that consume the bulk of LBP compensable care resources and for whom positive outcomes are possible but not frequent or substantial.

## Review

This descriptive review summarises current knowledge on prevalence, activity limitation (disability), care-seeking, natural history and clinical course, treatment outcome, and costs of low back pain (LBP). Reports of the epidemiology of LBP in primary care were identified through electronic searches of Medline, Cinhahl, Embase, Psychlit, and AMED from inception until October 2004. An example of the search strategies used is attached as Additional file 1. The search also included checking the reference lists of retrieved papers.

### Prevalence

Reviews of the literature describing LBP point prevalence in the developed world have produced variable estimates of prevalence rates [1,2]. In the studies deemed by Looney and Stratford to be methodologically superior, the LBP point prevalence was estimated to be 6.8% in North America, 12% in Sweden, 13.7% in Denmark, 14% in the United Kingdom, 28.4% in Canada, and 33% in Belgium [2]. The size of the difference between the North America LBP point prevalence estimated by Deyo and Tsui-Wu at 6.8% [3] and that of Canada at 28.4% [4] illustrates the variability attributable, in unknown proportion, to sample and sampling differences. In a review of world prevalence data, Volinn [5] suggested that there were lower rates of prevalence in developing countries than in developed countries, but did not determine whether differences reflect demographic, cultural or research method factors.

Walker [6] conducted a systematic review of the Australian LBP prevalence literature 1966–1998, and also concluded that the true prevalence of LBP in Australia remained confounded by methodological flaws in previous studies. Walker [7], subsequently surveyed 3000 Australian adults using contemporary epidemiological methods, and estimated the point prevalence of LBP at 25.5%, six-month period prevalence at 64.6% and lifetime prevalence at 79.2%. The retrospective one-year first incidence of LBP in the sample was 8.0%. These data suggest that LBP is common in the Australian population, with four out of five adults experiencing LBP in their life and approximately one in 12 experiencing a new episode of LBP over a 12-month period. A large difference between the point prevalence and the six-month prevalence of LBP in Walker's data is also seen in other epidemiological studies [8] and probably reflects the fluctuating, episodic nature of most LBP. This review did not uncover evidence of gender differences in LBP prevalence in adults sampled from the USA [3] Canada [4], Nordic countries [9] and Australia [7], nor in a Finish sample of children and adolescents [10].

The prevalence of LBP in children is low (1%-6%) [10] but increases rapidly (18%–50%) in the adolescent population [10-12]. The prevalence of LBP peaks around the end of the sixth decade of life. For example, in a prospective 12-month study of 4501 adults in the South Manchester region of the United Kingdom [8], the age distribution of LBP was unimodal, with the peak prevalence occurring in those aged 45 to 59 years old. This is similar to USA epidemiological data describing the peak point prevalence, period prevalence and lifetime prevalence all within ages 55 to 64 years [3]. Though some age-specific back pain cost data show a bimodal distribution with a peak for women over 75 years of age [13], it is likely that this does not represent an increase in the prevalence of non-specific back pain but the prevalence of serious pathology (including compression fracture).

Though LBP treatment and compensation costs have risen markedly over the last three decades [14-16], this may be more the product of case management and cultural attitudes regarding liability and compensation, than changes in either LBP prevalence or LBP activity limitation. There is no compelling biological argument as to why LBP should be increasing in prevalence. Prevalence rates, when measured annually using consistent methods, have shown no change in a Nordic population over a 15-year period [17]. There also is evidence that claim rates for occupational LBP appear to be decreasing in the USA [18], though the relationship of this to prevalence rates is not clear and may also represent an attitudinal change to compensation. Temporal variation in LBP reporting, medical investigation, litigation and compensation may reflect change in societal responses to this common condition rather than any change in LBP prevalence.

### Activity limitation (Disability)

In the USA, for people aged 45 years or less, LBP is the most frequent cause of activity limitation [19]. In Walker's data [7], over the previous 6-month period 42.6% of a sample of the Australian adult population reported experiencing low intensity LBP and low associated limitations of activity. A further 10.9% reported experiencing high intensity LBP, but also with low activity limitation. In contrast, an additional 10.5% reported experiencing high intensity LBP with high activity limitation. Though a common problem, it would appear that most LBP in Australia is of low intensity and results in low activity limitation. However, about one in 10 Australian adults have had activity limitation as a result of LBP in the past six months severe enough to result in significant time off from usual activities (Mean time off work = 1.6 months, median 18 days). These data are very similar to the 6-month LBP intensity and activity limitation data of a Canadian adult sample [4]. Though there was no gender difference in prevalence of activity limitation or participation restriction in an Australian LBP sample [7], women were twice as likely to report severe activity limitation in a Canadian sample [4].

### Care-seeking

In Walker's data [20], of those Australian adults who experienced LBP over the previous 6-month period, 44.3% sought health care for this condition. This was 28.6% of the total sample. Those seeking care had a greater fear that LBP could impair their life in the future and had higher pain levels than those who did not seek care. Carey et al [21] found that in a sample from North Carolina USA, 61% of recent-onset (<12 weeks) LBP sufferers sought care during their most recent episode. Those seeking care were likely to have more intense pain, leg pain, or a pain onset at work, than those who did not seek care. In a 1995 Australian survey, of those reporting back problems, 46% sought treatment [22]. In summary, about one in two people who experience LBP seek health care during an episode, and they tend to be those experiencing more severe pain, more distal pain, work-related pain or who are more fearful about what the pain might mean.

This review of the LBP epidemiologic evidence found only two studies examining gender differences in care-seeking by those with LBP. In a South Manchester study [8] there was a small gender difference in the frequency of general medical practice consultation for LBP, (mean 7.0% for women, 5.5% for men), but it is unclear whether real gender differences exist or reflect sampling error as the statistical significance of this difference was not reported. However, reinforcing the common perception that women display a greater willingness to seek care for health issues, in an Australian study Walker [20] found women more likely to seek care for LBP (adjusted odds ratio 1.7, 95%CI 1.3 to 2.2).

The most common clinicians consulted for back pain in North America are chiropractors, general medical practitioners and orthopaedists [3,23-25]. In Australia, the most common clinicians consulted for LBP are chiropractors, general medical practitioners, massage therapists, and physiotherapists [20]. People experiencing more severe pain [21,24], who have co-morbidity [24], and women [21] are more likely to consult medical practitioners rather than practitioners in other disciplines.

LBP is a sizeable proportion of casemix for some primary-contact disciplines. Physiotherapy LBP casemix has been estimated to be 25% [26] and 45% [27], depending on the clinical and cultural setting. Chiropractic LBP casemix has been estimated to be 41% in two Australian studies [28,29]. Back pain is the ninth most common presentation in Australian general medical practice [30], contributing between 3.8% [30] and 7.1% [31] of presenting complaints.

Clinicians may choose from a plethora of treatment options, and there are a number of quality evidence-based LBP practice guidelines that can inform those choices [19,32-35]. The extent to which primary-contact practice mirrors recommended practice is unknown [36]. The six most common types of treatment received by Australian adults when seeking care for LBP are back exercises/stretching, massage, spinal manipulation, prescribed medication, non-prescription medication, and bed rest [20]. The lack of knowledge regarding the etiology of most LBP and the lack of a coherent LBP treatment model with cross-discipline acceptance, results in highly varied LBP management strategies being implemented across and within primary-contact disciplines [37-39]. This can result in patient confusion and dissatisfaction [39].

### Natural history and clinical course

Von Korff [40] defined natural history as the development of a condition in the absence of treatment, and defines clinical course as its development in the presence of treatment. Studies of the 'natural history' of LBP are potentially compromised by the health care received by any study population, as it is not ethical to prohibit treatment to patients in order to observe the natural history. As there is evidence that specific conservative therapy, (for example, exercise or manipulation [19,33,41,42]) changes the course of an episode of LBP, it is not clear whether studies of the clinical course of people with LBP receiving treatment gives a trustworthy indication of the natural history.

Data describing the clinical course of LBP are also affected by variations in data collection methods, with higher quality studies including independent follow-up for at least 12 months after the onset of a LBP episode. Some reports describe a lack of patient care-seeking from a particular primary-contact practitioner as synonymous with recovery [43], but this approach suffers because people may cease seeking help for a number of reasons. Furthermore, reports of compensation patients, where return-to-work or the ceasing of wage supplementation is the only outcome measure, may not accurately describe the clinical course of LBP in the broader community due to factors affecting reporting, population bias, the complexity of factors that affect return-to-work, and the insensitivity of these outcome measures to LBP recurrence, residual pain and residual activity limitation. Given these considerations, it is reasonable to propose that complete recovery is not synonymous with return-to-work. In addition, up to 60% of injured workers are unable to sustain their initial return-to-work [44], which limits the information about the clinical course of LBP when data collection is confined to initial return-to-work. It is likely that a perspective of LBP derived from research that focuses on the outcome measures of return-to-work and claims management, will be different from a perspective derived from the study of symptom resolution and restoration of all activity (both vocational and non-vocational).

Recent systematic reviews of the clinical course of LBP [45,46] indicate that rapid improvements occur in the first three months post-onset, but that improvements are gradual thereafter. At 6 months post-onset, 16% (range 3–40%) of patients initially off-work remain off-work, and at 12 months post-onset, 62% (range 42–75%) still have pain. Within 12 months of onset, recurrences of both pain (60%, range 44–73%), and recurrences of work absence (33%, range 26–37%) [45] are common.

Ninety percent of the patients who experienced LBP in the South Manchester study [47] ceased consulting their general medical practitioner regarding these symptoms within three months. However, when subsequently interviewed, 79% at three-month follow-up and 75% at 12-month follow-up had not fully recovered (defined as VAS pain score < 2, Hanover Disability Score > 90%). Croft et al [48] recommend revising the view of recent-onset LBP as being self-limiting with only a small proportion that becomes persistent (>12 weeks), to a model of LBP as an essentially persistent condition, characterised by frequent episodes of symptoms interspersed with periods of relative freedom from pain and activity limitation. This recommendation has also been made in other reviews of the clinical course of LBP [34,49,50].

The group of recent-onset LBP patients who remain in intense pain and have substantial activity limitation at 12-months post-onset tend to be the cohort who also remain off-work at that time. However, Watson et al. [51] found that 12-months post-onset, whereas only 0.65% of those experiencing first-onset LBP were still off-work, 4.5% of those who were experiencing recurrences of pre-existing LBP still remained off-work. Recurrence therefore appears to increase the risk of not returning to work (relative risk 6.9). Studies from a number of national and vocational settings indicate that the longer workers remain off-work the lower the probability of them ever returning to work [50].

Although patients with persistent LBP are commonly thought to have a poor prognosis, there are few data describing their long-term outcomes. A Dutch group of patients with persistent LBP were followed for seven years and measures of pain, activity limitation, spinal mobility, and movement-related pain were repeatedly recorded. At the beginning of the study, the mean duration of back pain for the group was 5.4 years (SD 3.6). At three years post-initial measurement (n = 31), statistically significant improvements were found in pain and activity limitation scores, while lumbar spine mobility decreased [52]. At seven years post-initial measurement (n = 22), spinal mobility was unchanged from the three-year level, but further statistically significant improvements in activity limitation and movement-related pain had occurred [53]. These data suggest that once established, persistent LBP does not lead to progressive increases in pain and progressive increases in activity limitation. However, the mean scores for the variables measured were around 50% at the beginning of the study and did not improve over the study period by more than 15%. These data encourage the hypothesis that persistent LBP tends to stabilise and improve a little and slowly in the long-term. Data were obtained from a small sample and the hypothesis warrants testing on a larger sample.

A clinical feature of LBP and a dilemma for LBP research measurement is the recurrent, episodic nature of LBP, as it confounds conclusions based on measurements taken at a set point in time. This has led to recommendations that instead of data indicating numbers remaining off-work at a set point in time, such as 12-months after onset, measures such as total number of days off-work over a 12-month period may be more informative. The same principle can be applied to other dimensions of the LBP experience, for example, measuring the number of days in pain over a period, instead of those still in pain at the end of the period [54]. This fluctuating clinical course of LBP with incomplete resolution has led some authors to suggest that the distinction between acute (recent-onset) and chronic (persistent) LBP is clinically irrelevant [55]. In summary, the clinical course of recent-onset LBP is that patients are likely to recover from their presenting episode, most will still have some symptoms at 12 months, many will experience relapses, and a few will not improve much at all despite treatment.

### Treatment outcomes

There are now many randomised controlled trials (RCT) of interventions in both recent-onset and persistent LBP. These trials vary greatly in subject inclusion/exclusion criteria, outcome measures, blinding, concealment, analysis techniques and other research design features. This diversity, combined with the poor quality of many RCTs, has made data synthesis difficult, and resulted in few meta-analyses. Most synthesis of LBP intervention data has been via systematic review. Systematic reviews also vary in methodological quality and in the papers selected for inclusion. Furthermore, even reviews that broadly cover the same literature are subject to author interpretation, and many reach conflicting conclusions regarding intervention effectiveness [56,57]. Reviews with higher methodological rigour tend to report more negative or uncertain conclusions about the effects of interventions for LBP [58].

There are a number of exhaustive reviews of the efficacy of interventions in recent-onset LBP [19,33,34,42,59]. There are also a number of national clinical guidelines for the management of LBP that have been based on comprehensive literature searches [19,33,34,59-66]. Their recommendations regarding positive interventions for recent-onset LBP can be summarised as: patient education and reassurance, medication (Paracetomol, NSAIDs, muscle relaxants, opioids), some forms of exercise, manual therapy (manipulation, mobilisation), and discouragement of bed rest [36].

In a study of reviews of conservative treatment for persistent LBP, Furlan et. al. [57], summarised the results of 109 systematic reviews. The interventions included medication (analgesics, antidepressants, epidural and facet injections, muscle relaxants, NSAIDs, and opioids), education/behavioural (back schools, bed rest, cognitive/behaviour, couple therapy, multidisciplinary teams), and physical treatments (acupuncture, exercise, laser, orthoses, spinal manipulation, TENS, traction). The summaries produced mostly negative or conflicting findings. They concluded that the only interventions associated with positive patient outcomes were muscle relaxants, opioids, and interventions provided by multidisciplinary teams.

### LBP costs

The direct financial costs of back pain are health care costs, and indirect costs are production losses to industry and injury impact on insurance costs. Estimates of the indirect costs vary depending on the econometric model chosen. Annual back pain costs have been estimated for Australia [67], the United Kingdom [68] and USA [14], and are summarised in Table 1. Across these countries, the direct costs of back pain represent between 0.19% and 0.42% of GDP, and between 1.65% and 3.22% of all health expenditure.

**Table 1:** Annual back pain financial costs.

**Annual back pain costs**					
	Direct costs (millons)	Allied health costs (millons)	Indirect costs^# ^(millons)	GDP*	Health expenditure

Australia (1991)	AU$1,020			0.22%	1.65%
		AU$724		0.16%	1.17%
			AU$2,000–$8,000	0.43%–1.72%	
United Kingdom (1998)	£1,632,000			0.19%	2.78%
			£1,068,000–£5,018,000	0.12%–0.58%	
USA (1990)	US$24,300			0.42%	3.22%

^#^Estimates of indirect costs vary depending on the econometric models used.

*GDP = Gross Domestic Product (for year of study).

During 1993/4, in an Australian population of 19.5 million people, there were 3.6 million medical consultations and 2.9 million prescriptions for back pain [13]. However, across the countries in which it has been studied, the majority of compensable LBP costs are generated by a small proportion of claimants. For example, data from the Quebec Workers Compensation System showed that the 8% of claimants who were absent from work for more than six months were responsible for 73% of the medical costs, and 76% of the compensation costs [69].

Direct costs to the health care and compensation systems, and indirect costs to industry do not include the non-financial costs to the patient and his/her family. These non-financial costs include lost participation in domestic, family, and social activities.

## Conclusion

LBP is a common problem affecting both genders and most ages, for which about one in four adults seeks care in a six-month period. It results in considerable direct and indirect costs, and these costs are financial, workforce and social. Care-seeking behaviour varies depending on cultural factors, the intensity of the pain, the extent of activity limitation and the presence of co-morbidity. Care-seeking for LBP is a significant proportion of caseload for some primary-contact disciplines. Most recent-onset LBP episodes settle but only about one in three resolves completely over a 12-month period. About three in five will recur in an on-going relapsing pattern and about one in 10 does not resolve at all. The cases that do not resolve at all form a persistent LBP group that consume the bulk of LBP compensable care resources and for whom positive outcomes are possible but not frequent or substantial.

## Authors' contributions

PMK conceived of the study, participated in its design, located and selected studies, extracted and interpreted the data, wrote the paper, and approved the final manuscript. JLK conceived of the study, participated in its design, interpreted the data, and revised and approved the final manuscript.

## Supplementary Material

Additional File 1 It contains the strategy used for the OVID Medline Search. Click here for file
