# Supplementary material for: The epidemiology of low back pain in primary care
Source: Chiropr Osteopat. 2005 Jul 26;13:13. doi: 10.1186/1746-1340-13-13 (PMC1208926; doi:10.1186/1746-1340-13-13)
Supplement: Additional File 1 — It contains the strategy used for the OVID Medline Search. [file 1746-1340-13-13-S1.doc]

### Additional file 1 – Example systematic review search strategy

OVID Medline Search Strategy

1 exp BACK PAIN/ or BACK PAIN.mp.

2 exp LOW BACK PAIN/cl, pp, di, pc, px, ra, ep, rh, et, th [Classification, Physiopathology, Diagnosis, Prevention & Control, Psychology, Radiography, Epidemiology, Rehabilitation, Etiology, Therapy]

3 exp "SPRAINS AND STRAINS"/ and exp SPINE/

4 exp INTERVERTEBRAL DISK DISPLACEMENT/ or INTERVERTEBRAL DISK DISPLACEMENT.mp.

5 SCIATICA/ or sciatica.mp.

6 BACK PAIN.ti,ab.

7 BACK STRAIN.ti,ab.

8 LOW BACK PAIN.ti,ab.

9 SIMPLE BACK PAIN.ti,ab.

10 NON-SPECIFIC BACK PAIN.ti,ab.

11 LOW BACK SYNDROME.ti,ab.

12 LOW BACK DYSFUNCTION.ti,ab.

13 LUMBAR PAIN.ti,ab.

14 BACKACHE.ti,ab.

15 LUMBAGO.ti,ab.

16 1 or 2 or 3 or 4 or 5 or 6 or 7 or 8 or 9 or 10 or 11 or 12 or 13 or 14 or 15

17 limit 16 to (HUMAN and ENGLISH language)

18 exp CASE-CONTROL STUDIES/ or exp CLINICAL TRIALS/ or exp RESEARCH DESIGN/ or exp RANDOMIZED CONTROLLED TRIALS/ or exp PROSPECTIVE STUDIES/

19 exp REVIEW LITERATURE/

20 exp META-ANALYSIS/

21 exp PROSPECTIVE STUDIES/mt [Methods]

22 NON EXPERIMENTAL STUD$.mp.

23 (CLIN$ adj25 TRIAL$).ti,ab.

24 ((SING$ or DOUBL$ or TREBL$ or TRIPL$) adj25 (BLIND$ or MASK$)).ti,ab.

25 PLACEBO$.mp. or RANDOM$.ti,ab. [mp=title, abstract, cas registry/ec number word, mesh subject heading]

26 (CONTROL$ or PROSPECTIV$ or VOLUNTEER$).ti,ab.

27 OBSERV$.ti,ab.

28 (CASE adj25 (CONTROL$ or SERIES)).ti,ab.

29 (SYSTEMAT$ adj25 REVIEW).ti,ab.

30 (LITERATURE adj25 REVIEW).ti,ab.

31 RANDOMIZED CONTROLLED TRIAL.pt.

32 CONTROLLED CLINICAL TRIAL.pt.

33 RANDOMIZED CONTROLLED TRIALS.sh.

34 RANDOM ALLOCATION.sh.

35 DOUBLE-BLIND METHOD.sh.

36 SINGLE-BLIND METHOD.sh.

37 CLINICAL TRIAL.pt.

38 18 or 19 or 20 or 21 or 22 or 23 or 24 or 25 or 26 or 27 or 28 or 29 or 30 or 31 or 32 or 33 or 34 or 35 or 36 or 37

39 17 and 38

40 exp DIAGNOSTIC ERRORS/ or exp DIAGNOSTIC IMAGING/ or exp "DIAGNOSTIC TECHNIQUES AND PROCEDURES"/ or exp DIAGNOSTIC TESTS, ROUTINE/

41 (SIGNS AND SYMPTOMS).mp. [mp=title, abstract, cas registry/ec number word, mesh subject heading]

42 ACTIVITIES OF DAILY LIVING/cl, px, sn, mt [Classification, Psychology, Statistics & Numerical Data, Methods]

43 exp DISABILITY EVALUATION/mt, cl, st, sn [Methods, Classification, Standards, Statistics & Numerical Data]

44 exp AFFECTIVE SYMPTOMS/ or AFFECTIVE SYMPTOMS.mp.

45 exp ANXIETY/ or exp DEPRESSION/ or exp BEHAVIOR/

46 exp PSYCHOLOGICAL TESTS/ or PSYCHOLOGICAL TESTS.mp.

47 exp DECISION MAKING/ or CLINICAL DECISION MAKING.mp. or exp DECISION SUPPORT TECHNIQUES/ or exp DIAGNOSIS/ or exp PRIMARY HEALTH CARE/

48 exp RADIOGRAPHY/cl, px, di, st, sn, ut, mt [Classification, Psychology, Diagnosis, Standards, Statistics & Numerical Data, Utilization, Methods]

49 exp TOMOGRAPHY/ or exp TOMOGRAPHY, X-RAY/ or exp TOMOGRAPHY, X-RAY COMPUTED/ or TOMOGRAPHY.mp.

50 exp MAGNETIC RESONANCE IMAGING/ or MAGNETIC RESONANCE IMAGING.mp.

51 exp ULTRASONOGRAPHY/ or ULTRASONOGRAPHY.mp.

52 (TEST$ or SIGN$ or SYMPTOM$ or INSTRUMENT$ or DIAGNOS$ or MEASUR$ or FUNCTION$ or OUTCOME$ or CLASSIF$ or COMPARI$).ti,ab.

53 40 or 41 or 42 or 43 or 44 or 45 or 46 or 47 or 48 or 49 or 50 or 51 or 52

54 39 and 53

55 RECOVERY.mp.

56 exp NATURAL HISTORY/ or NATURAL HISTORY.mp. or exp PROGNOSIS/

57 RESPONSE.mp.

58 exp "OUTCOME ASSESSMENT (HEALTH CARE)"/ or exp TREATMENT OUTCOME/ or OUTCOME.mp.

59 PREDICTIVE UTILITY.mp. or exp PREDICTIVE VALUE OF TESTS/

60 TREATMENT RESPONSE.mp.

61 RECURRANCES.mp.

62 CLINICAL INDICATORS.mp.

63 OUTCOMES RESEARCH.mp.

64 (NATURAL adj25 HISTORY).ti,ab.

65 exp UTILIZATION REVIEW/ or exp REPRODUCIBILITY OF RESULTS/ or VALIDITY.mp. or exp PSYCHOMETRICS/ or exp RESEARCH/

66 (TREATMENT adj25 RESPONSE).ti,ab.

67 PROGNOS$.ti,ab.

68 OUTCOME$.ti,ab.

69 PREDICT$.ti,ab.

70 VALIDITY.ti,ab.

71 RECOVERY.ti,ab.

72 UTILITY.ti,ab.

73 RECURRANCE$.ti,ab.

74 55 or 56 or 57 or 58 or 59 or 60 or 61 or 62 or 63 or 64 or 65 or 66 or 67 or 68 or 69 or 70 or 71 or 72 or 73

75 54 and 74

76 SURGERY.mp. or exp SURGERY/

77 exp NEOPLASMS/ or CANCER.mp.

78 exp NEOPLASM METASTASIS/ or METASTASIS.mp.

79 exp INFLAMMATION/ or INFLAMMATION.mp.

80 exp ARTHRITIS, JUVENILE RHEUMATOID/ or exp ARTHRITIS, RHEUMATOID/ or RHEUMATOID.mp.

81 exp INFECTION/ or INFECTION.mp.

82 exp PREGNANCY/ or PREGNANCY.mp.

83 exp FRACTURES/ or FRACTURE.mp.

84 exp ARTHRITIS, PSORIATIC/ or PSORIATIC ARTHRITIS.mp.

85 exp GOUT/ or GOUT.mp.

86 exp REITER DISEASE/ or REITER'S DISEASE.mp.

87 exp DISKECTOMY/ or DISKECTOMY.mp.

88 (SURGER$ or CANCER$ or METASTAS$ or TUMOR$ or TUMOUR$ or NEOPLASM or INFLAMMAT$ or RHEUMATOID or INFECTION$ or PREGNAN$ or FRACTURE$ or GOUT or ANIMAL or DISKECTOMY or DISCECTOMY).ti,ab.

89 (REITER'S adj25 DISEASE).ti,ab.

90 76 or 77 or 78 or 79 or 80 or 81 or 82 or 83 or 84 or 85 or 86 or 87 or 88 or 89

91 75 not 90
